# The incidence of radicular groove on maxillary lateral incisors of Saudi population: CBCT evaluation

**DOI:** 10.1186/s12903-022-02616-1

**Published:** 2022-12-09

**Authors:** Sarah M. Alkahtany, Fatemah Alrwais, Asma Altamimi, Sundus M. Bukhary, Amani Mirdad

**Affiliations:** 1 Department of Restorative Dental Sciences, Division of Endodontics, College of Dentistry, King Saud University, PO Box 68004, 11527 Riyadh, Saudi Arabia; 2 Department of Dentistry, Division of Endodontics, King Saud Medical city, Ministry of Health, 12746 Riyadh, Saudi Arabia; 3 Department of Periodontics and Community Dentistry, College of Dentistry, King Saud University, PO Box 68004, 11527 Riyadh, Saudi Arabia

**Keywords:** Radicular groove, Palatal groove, Dental anomaly, Endodontics

## Abstract

**Background:**

The radicular groove (RG) is one of the developmental anomalies that is commonly found in maxillary incisors. The formation of radicular groove is initiated around the cingulum and can reach the root at different levels. The incidence of radicular grooves was reported in different countries but there was no published data about the incidence of RG in Saudi Arabia. Therefore, this study aimed to evaluate the incidence of radicular grooves on maxillary lateral incisors in the Saudi population using cone-beam computed tomography (CBCT).

**Methods:**

The dental records of 490 patients (N = 490) with CBCT images of maxillary anterior teeth were screened for inclusion criteria. Then 264 included cases were evaluated independently by two Endodontists. The evaluation was performed on CBCT images in the axial, sagittal, and coronal sections using Planmeca Romexis® software. The following data were recorded for each patient: Patients’ age and gender, radicular groove presence or absence, and if it is bilateral or unilateral. The type of radicular groove was recorded according to Gu’s classification (type I, II, or III).

**Results:**

The incidence rate of radicular grooves in maxillary lateral incisors was 4.9%. RG was found to be unilateral in 61.5% and bilateral in 38.5%. The majority of RG were classified as type I in 69.2%, followed by type II in 15.4%, and type III was found in 15.4%.

**Conclusion:**

4.9% of the Saudi population has RG in the upper lateral incisor. This anatomical variation is mostly present as type I on one side only (unilateral).

**Supplementary Information:**

The online version contains supplementary material available at 10.1186/s12903-022-02616-1.

## Background

The radicular groove (RG) was first described by Black in 1908, it is one of the developmental anomalies that is commonly found in maxillary incisors. The formation of RG is initiated around the cingulum and can reach the root at different levels [1–3]. The exact aetiology of the RG remains unclear. Previous studies reported that the RG formation might be due to genetic factors. During tooth formation, the inner enamel organ and Hartwig’s epithelial root sheath will be enfolded resulting in groove formation [4–6]. This groove was reported with different names in the literature such as palatal, palatal-gingival, Cingular-radicular, radicular-lingual, disto-lingual, and vertical developmental groove [7–10]. Embryological changes and developmental abnormalities, such as supernumerary teeth, missing teeth, peg-shaped incisors, and cleft lip and palate, may occur in a greater incidence in the maxillary anterior area [11–16]. Likewise, RGs are found in the palatal surface of the maxillary incisors, with a higher incidence in maxillary lateral incisors [3, 8, 12, 13, 17, 18].

RGs have been classified from different aspects. Bacic et al. classified RGs into three categories based on the groove’s location (mesial, distal, or mid-palatal) [8]. The location of the origin and the termination were considered in the Kogon classification [3]. In 2011, Gu categorized the RGs into three types based on the degree of severity on micro-computed tomography. The short groove in the coronal third of the root was classified as type I, the long shallow groove beyond the coronal third of the root was classified as type II, and the long deep groove beyond the coronal third of the root was classified as type III [19].

The shorter RGs (Type I) may be asymptomatic, however, the deeper grooves (Type II & III) are considered clinically significant as they promote the accumulation of bacterial plaque and calculus [4]. These grooves are not easily accessible and are difficult to be cleaned, leading to the development of localized progressive periodontal inflammation [8, 19]. This periodontal destruction will allow the bacteria to reach the pulp cavity rapidly through accessory canals or even through apical foramen resulting in secondary pulpal infection and subsequent periapical pathosis [20–22, 22–24].

Extraction used to be the only treatment option for teeth with RGs. Recently, more conservative treatment modalities were recommended and showed more favorable outcomes [4, 5, 7, 12, 25–27]. Mild radicular grooves might be treated with odontoplasty combined with periodontal treatment. Furthermore, shallow grooves could be sealed with restorative materials [28, 29].

However, in the deeper more complex grooves more interventions might be required such as root canal treatment, periodontal curettage, cauterization with or without guided tissue regeneration therapy, and intentional replantation. Extraction was always recommended for hopeless cases [7, 22, 28, 30–33].

Previous studies reported that the prevalence of RG ranged from 0.90 to 44.6%. This broad range of the reported studies can be affected by the differences in methodology, ethnicity, and region [2, 3, 8, 12, 13, 18, 34, 35]. Many approaches have been used to evaluate RGs. Clinical examinations and conventional radiographs were used to detect RGs, but they did not provide sufficient information about the groove extending below gingival tissue and alveolar bone. In some cases, surgical exposure is required for proper diagnosis. Thus cone-beam computed tomography (CBCT) could be an alternative effective conservative method [19, 22, 35–37]. CBCT offers non-invasive and accurate information to examine teeth morphology, root canal anatomy as well as RGs [2].

Many studies were published about the incidence of RGs in different countries and variable populations. However, no previous publication is concerned about the Saudi Arabian population. Therefore, this study aimed to evaluate the incidence of RG on maxillary lateral incisors in Saudi Arabia using cone-beam computed tomography (CBCT).

## Methods

This study was conducted in the College of Dentistry, King Saud University, Saudi Arabia. The study protocol was approved by the institutional review board (IRB No. E-20–5240). The dental records of 490 patients (N = 490) with CBCT images of maxillary anterior teeth were screened for inclusion criteria. All CBCT images were obtained with a CBCT machine (NewTom 5G1, QR, Verona, Italy) from patients referred to the radiology department with different problems such as complex endodontics conditions or evaluation of implants from 2016 to 2021. The voxel size range from 0.15 to 0.3 mm and the slice thickness is 1.0 mm. The study protocol of Arslan 2014 was followed in this retrospective study [2].

**Fig. 1:**
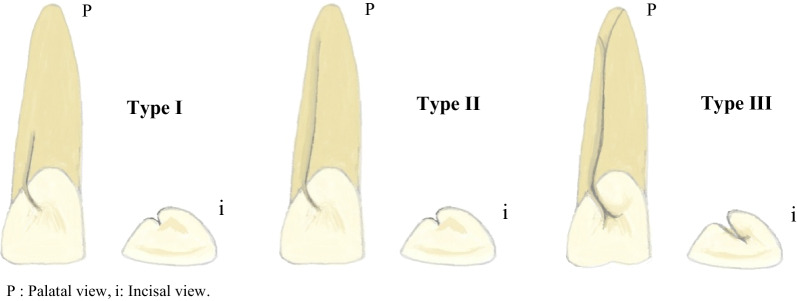
Illustration of Gu’s Classification based on the severity. Type I: short groove (not beyond the coronal third of the root). Type II: long and shallow groove (beyond the coronal third of the root). Type III: long and deep groove (beyond the coronal third of the root), associated with complex root canal system

The inclusion criteria were the presence of high-quality CBCT images and the presence of bilateral maxillary lateral incisors. Any case with extensive coronal restorations, root canal fillings, and posts, internal/external resorption, cleft lip, and palate, impacted teeth in the maxillary anterior region, and deep caries was excluded. Then included cases were evaluated independently by two Endodontists. The evaluation was performed on CBCT images in the axial, sagittal, and coronal sections using Planmeca Romexis® software. The following data were recorded for each patient: Patients’ age and gender, RG presence or absence, and if the RG is bilateral or unilateral. The type of RG was recorded according to Gu’s classification: type I, II, or III (Fig. 1) [19].

Agreement between evaluators was tested with Kappa statistics. Statistical analysis was performed by SPSS 24.0 version (IBM Inc., Chicago USA) statistical software. A Chi-square test was used to evaluate the incidence of RG, the location (unilateral or bilateral), and RG type (type I, II, or III).

## Results

According to Kappa statistics, there was a good agreement between the two evaluators (Kappa = 0.788) regarding the detection of RG on CBCT images. A total number of 490 patients with CBCT images of the maxillary anterior region were screened for inclusion criteria. Only 264 patients were included in this study, 199 patients (75.4%) were females and 65 patients (24.6%) were males, all between 18 and 80 years old (Additional file 1).

**Fig. 2:**
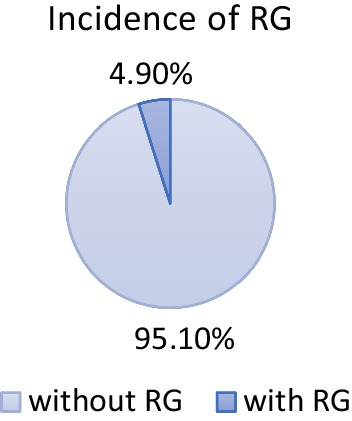
Incidence of radicular grooves on maxillary lateral incisors in 264 patients

**Fig. 3:**
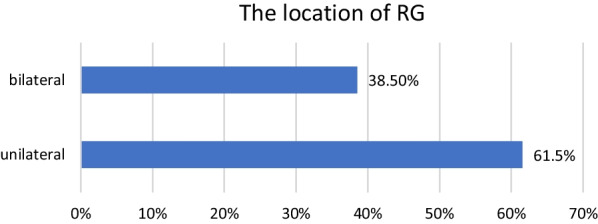
The location of RG in 13 patients

**Fig. 4:**
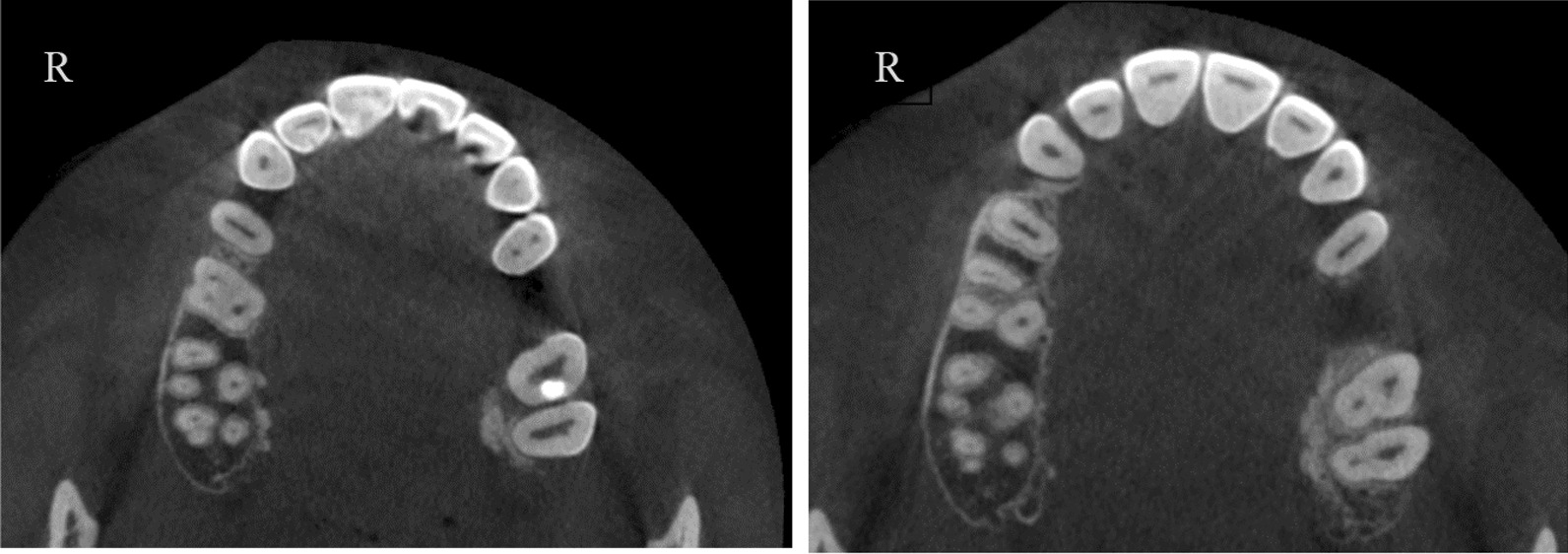
Type I bilateral RG: shallow RG can be seen on right and left sides at different levels

**Fig. 5:**
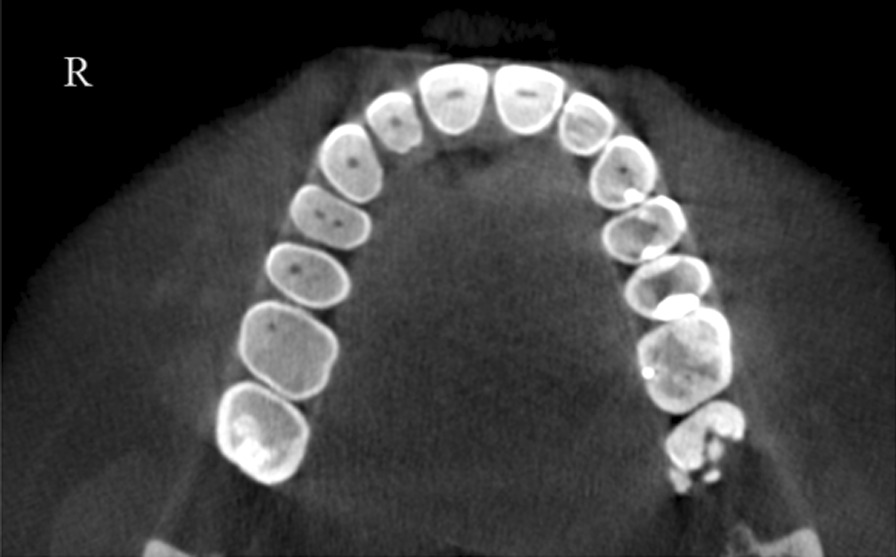
Type I unilateral: shallow RG on the right side only

**Fig. 6:**
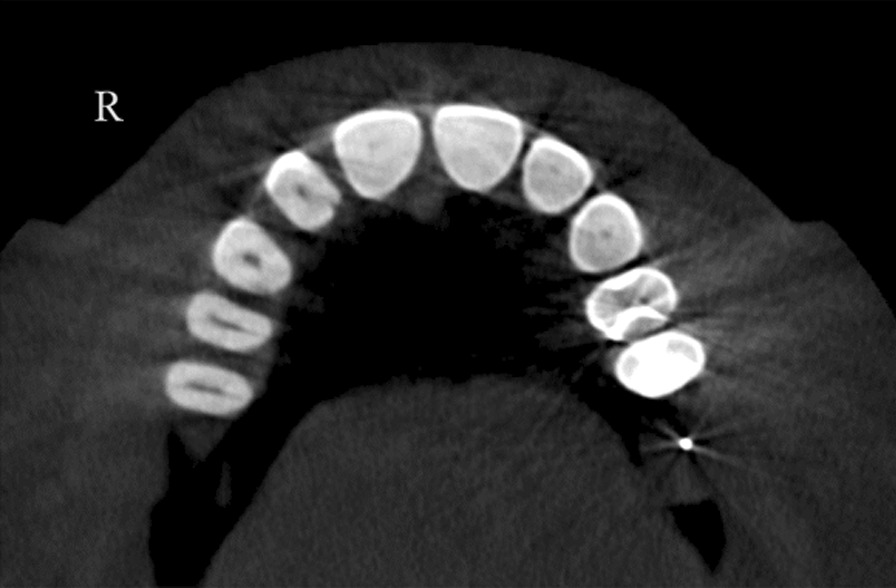
Type II unilateral: right side

**Fig. 7:**
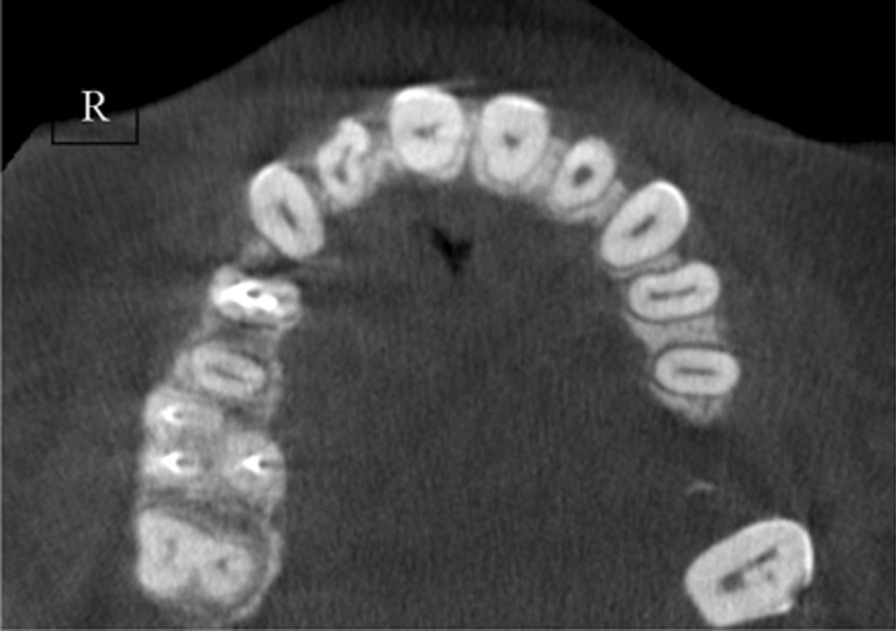
Type III unilateral: deep groove on the right side. (severe form)

A total of 13 patients (10 females and 3 males) were detected with RG based on CBCT evaluation. The total incidence rate of RGs in maxillary lateral incisors was 4.9% (Fig. 2). The incidence was 5% for females and 4.6% for males with no significant difference between different genders (*P* > 0.05). RG was found to be unilateral in 61.5% and bilateral in 38.5% (Fig. 3). The majority of RG were classified as type I (Figs. 4, 5) in 69.2%, followed by type II (Fig. 6) in 15.4%, and type III (Fig. 7) was found in 15.4% (Table 1).

**Table 1:** Different classifications and locations of 13 Radicular groove cases out of 264 patients evaluated from CBCT images of maxillary lateral incisors

RG classification	Unilateral	Bilateral	Total
Type I	4 (30.7%)	5 (38.5%)	9 (69.2%)
Type II	2 (15.4%)	0 (0.0%)	2 (15.4%)
Type III	2 (15.4%)	0 (0.0%)	2 (15.4%)
Total	8 (61.5%)	5 (38.5%)	13 (100%)

## Discussion

Periodontal destruction and secondary endodontic infections might result from the presence of RGs grooves. Those grooves are located on the palatal aspect of maxillary incisors [24]. A proper diagnostic test and evaluation of clinical signs play an important role in successful endodontic diagnosis and treatment. Previous studies assessed the incidence of RGs in different populations and their results vary greatly between 2.2 and 30%. Different methodologies were used to assess the prevalence of RGs such as photographs, and micro-computed tomography in vitro. Clinical examination, radiographic examination, and CBCT were used in vivo. The results vary [2, 3, 8, 12, 13, 34, 35, 38].

New modalities of imaging such as cone-beam computed tomography (CBCT) provide three-dimensional (3D) high-resolution accurate images, with more information about the internal canal anatomy and external root details including the radicular groove extension [11]. Although CBCT has a lot of advantages, it has some limitations including the higher dose of radiation and possible artifact generation [39]. it is known that conventional periapical radiograph produces two-dimensional Images with inevitable geometric distortion and noise which affect the examination of root canal morphology accurately. Thus, we choose CBCT in the present study to assess the presence of radicular grooves on maxillary lateral incisors [40–43].

The present study showed that 4.9% of upper lateral incisors have RGs, this was slightly different than previous CBCT investigations. In the Turkish population, Arslan et al. reported an incidence of 2.3% & Aksoy et al. reported an incidence of 2.2%. However, the incidence of RG was 7.3% in the Indian population as reported by Varun et al. This difference in the incidence of RGs might be due to differences in sample ethnic & genetic factors. Furthermore, the sample size may affect the incidence rate in different studies. The current data revealed that most detected RGs were unilateral, and type I RG was the most common classification detected. This is in agreement with previous reports [2, 44, 45].

Our CBCT retrospective investigation detected only 2 patients with unilateral Type II RGs and 2 patients with unilateral Type III RGs. This might be due to the fact that deeper RGs are commonly associated with periodontal and endodontic symptoms, which will be indicated for extraction and endodontic treatment [34]. Patients with missing or endodontically treated maxillary lateral incisors were excluded from our study. Therefore, we might missed those deeper grooves (Type II & III), and only sound lateral incisors were included which are usually associated with the shallower groove (Type I).

The majority of the patients with RG were females because 75.4% of our sample were females. This result might be because females seek dental treatment more than males [46]. However, there was no significant difference in the incidence of RG between males and females. This is in agreement with Aksoy et al. [45].

The current retrospective study had some limitations, a large number of CBCT images were excluded due to the presence of artifacts resulting from adjacent restorations or crowns. Those artifacts will prevent the detection of RG, we might exclude some cases with RGs that we were unable to detect. Moreover, a lot of cases have been excluded that had RCT in anterior teeth which might be due to infection caused by RGs. Finally, we do not recommend using CBCT as the sole method to detect RG due to its limitations. A careful clinical examination should be performed before any radiographic assessment to precisely diagnose this anomaly.

Our findings indicated that RGs are not rare in our Saudi Arabian community. Therefore, clinicians should always consider the presence of this groove and other anatomical variations during clinical examination and treatment planning. RGs are clinically significant as they promote the accumulation of plaque and calculus leading to periodontal and pulpal pathosis.

## Conclusion

4.9% of the Saudi population has RG in the upper lateral incisor. This anatomical variation is mostly present as type I on one side only (unilateral). During clinical examination and treatment planning, clinicians should always consider the presence of RG and other anatomical variations.

## Supplementary Information

**Additional file 1:** The raw data collected from the CBCT of 264 patients including: patient’s age, gender, presence of radicular groove, is it unilateral or bilateral, and the classification of radicular groove.

## Data Availability

All generated data used and/or analysed during the current study were included in the supplementary infromation files.
